# Characterization of AiiK, an AHL lactonase, from *Kurthia huakui* LAM0618^T^ and its application in quorum quenching on *Pseudomonas aeruginosa* PAO1

**DOI:** 10.1038/s41598-018-24507-8

**Published:** 2018-04-16

**Authors:** Weiwei Dong, Jie Zhu, Xiang Guo, Delong Kong, Qi Zhang, Yiqing Zhou, Xiaoyang Liu, Shumiao Zhao, Zhiyong Ruan

**Affiliations:** 1 State Key Laboratory of Agricultural Microbiology and College of Life Science and Technology, Huazhong Agricultural University, Wuhan, 430070 China; 2 Institute of Agricultural Resources and Regional Planning, CAAS, Beijing, 100081 China; 3 College of life science, Xinjiang Normal University, Xinjiang, 830046 China

## Abstract

*N*-Acyl homoserine lactones (AHLs) act as the key quorum sensing (QS) signal molecules in gram-negative bacteria, which coordinates gene expression and then activates various processes, including biofilm formation and production of virulence factors in some pathogens. Quorum quenching (QQ), which is the inactivation of the signal molecules by means of enzymatic degradation or modification, inhibits the processes of QS rather than killing the pathogens and is a promising antipathogenic strategy to control the bacterial pathogens. In this study, an AHL lactonase gene (named *aiiK*) was cloned from *Kurthia huakuii* LAM0618^T^ and the AHL lactonase AiiK was expressed by *Escherichia coli*. AiiK exhibits a variable substrate spectrum and efficient degradation of the AHL compounds. The enzyme assays demonstrated that AiiK behaves as an AHL lactonase that can hydrolyze the lactone bond of the AHLs. The total hydrolytic efficiency of AiiK for C_10_-HSL is 3.9 s^−1^·mM^−1^. AiiK can also maintain 20% activity after 12 h incubation at 37 °C and demonstrate great resistance to α-chymotrypsin, trypsin, and protease K. Furthermore, AiiK significantly inhibits the biofilm formation and attenuates extracellular proteolytic activity and pyocyanin production of *Pseudomonas aeruginosa* PAO1, which indicates the potential application of AiiK as a biocontrol agent or an anti-pathogenic drug.

## Introduction

Quorum sensing (QS) is a common and important bacterial communication mechanism that monitors the population density of bacteria and controls the expression of specialized structural gene sets by using specific receptors that can sense the accumulation of signal molecules. QS systems regulate a variety of functions, including biofilm maturation, bioluminescence, sporulation, production of secondary metabolites, and competence for DNA uptake^1^. A diverse array of diseases are caused by a range of pathogens that utilize these QS systems, such as the opportunistic pathogen bacteria *Pseudomonas aeruginos* can develop persistent complicated biofilm associated infections and stimulate the resistance to antibiotics, and the plant pathogen bacteria *Erwinia carotovora* can produce pectolytic enzymes that hydrolyze pectin between individual plant cells, which causes a disease plant pathologists term bacterial soft rot^1^.

*N*-Acyl homoserine lactones (AHLs) act as key QS signal molecules in gram-negative bacteria. A representative AHL molecule comprises a homoserine lactone and an acyl chain containing an even number of carbon chains or a carbonylation at the C-3 position^2^. Quorum quenching (QQ), which is the inactivation of these signaling molecules by enzymatic degradation or modification, interferes with the QS systems and inhibits the processes of QS^3^. QQ enzymes, including AHL lactonases, AHL acylases, and AHL oxidoreductases, play an important role in QQ^1,4–7^. AHL lactonases belong to the metallo-β-lactamase superfamily and catalyze the opening of homoserine lactone bonds in AHLs^8–10^. Unlike AHL acylases and AHL oxidoreductases, AHL lactonases present variable substrate spectra and efficient degradation of AHLs signals^5,7,11^.

To date, over 20 QQ enzymes have been reported and the sources of which range from bacteria to fungi and even mammals^12,13^. QQ enzymes have been considered as novel biocontrol agents, particularly in antivirulence and antibacterial. The first reported AHL lactonase, AiiA, was identified in *Bacillus* sp. strain 240B1 and heterologously expressed in *E. carotovora* strain SCG1. AiiA significantly attenuated the pathogenicity of the *E. carotovora* strain SCG1 on many vegetables, such as carrot, potato, cauliflower, and so on^4^. The heterologous expression of AHL lactonase AiiM by *Escherichia coli* quenched the virulence in *Pectobacterium carotovorum* subsp. *carotovorum*^10^. MomL, a novel type of AHL lactonase from flounder mucus-derived *Muricauda olearia*, significantly alleviated the production of virulence factors in *P. aeruginosa* PAO1^14^. HqiA, a novel AHL-degrading enzyme type has AHL lactonase activity, interfered the swarming motility and production of maceration enzymes of *P. carotovorum*^15^. Therefore, the AHL lactonases provide a promising prospect to be applied as biocontrol agents.

In our previous studies, *K. huakuii* LAM0618^T^ was isolated from biogas slurry^16^. Genomic data^17^ revealed that *K. huakuii* LAM0618^T^ contains a putative protein sequence annotated as an “*N*-acyl homoserine lactonase”. Bioinformatic analysis suggested that it may represent a potential AHL lactonase. In the current work, the putative *aiiK* gene from *K. huakuii* LAM0618^T^ was cloned and heterologously expressed in *E. coli* based on method of genome-mining. The physicochemical properties of the purified recombinant AiiK protein (AiiK) and its application in QQ on *P. aeruginosa* PAO1 were investigated.

## Results

### Sequence analysis and identification of AiiK

**Figure 1:**
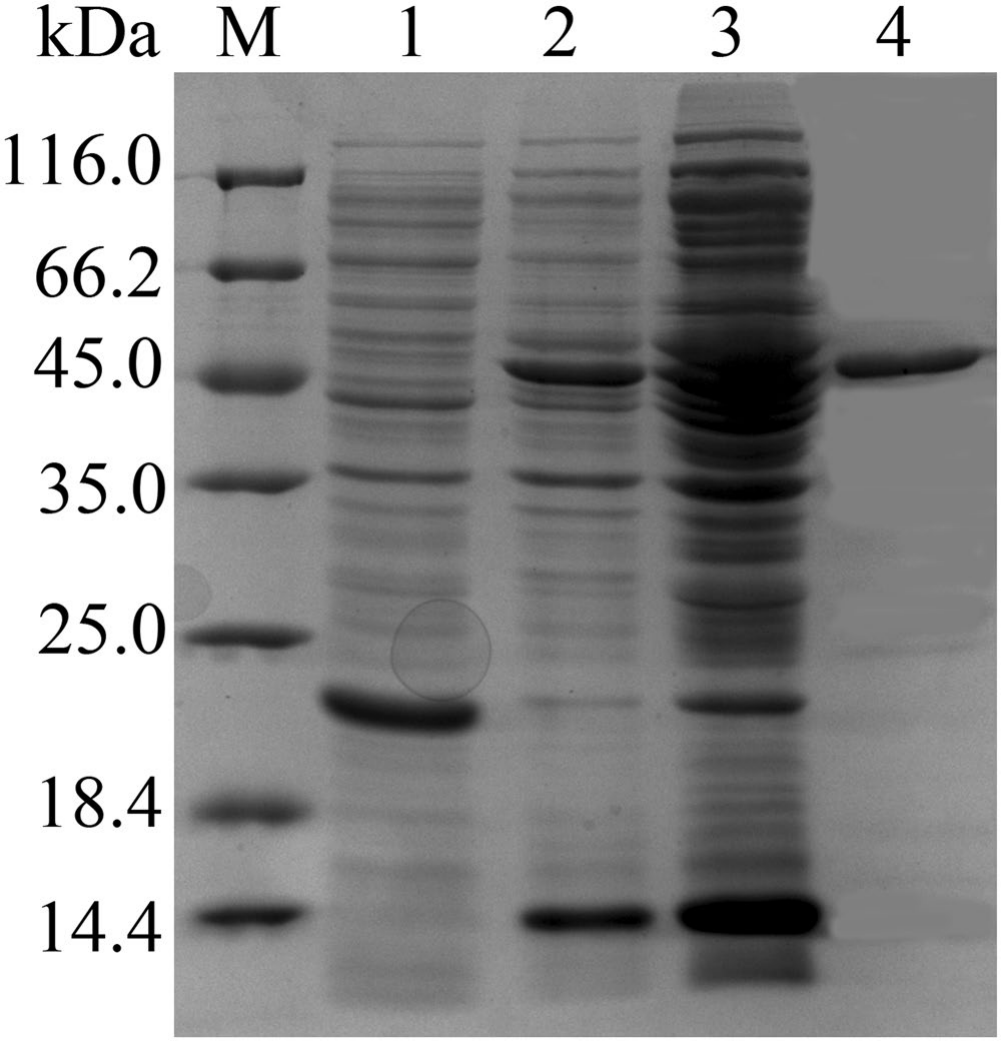
SDS-PAGE analysis of the purified AiiK. M: molecular weight marker; Lane 1: pET32a vector plasmid (control); Lane 2: supernatant of the sonication product; Lane 3: precipitate of the sonication product; Lane 4: AiiK purified via Ni–NTA.

**Figure 2:**
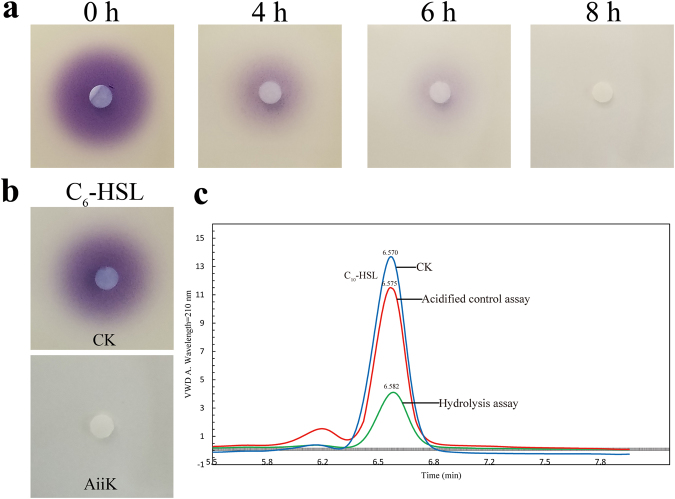
(**a**) AHL-degrading activity bioassays *in vivo*. Subcultures of *E. coli* BL21 (DE3) cells harboring pET32a-*aiiK* were mixed with 10 μM C_6_-HSL and incubated at 37 °C for 0 h, 4 h, 6 h, 8 h, respectively. (**b**) AHL-degrading activity bioassays *in vitro*. The purified AiiK (4 μg/mL) were mixed with 10 μM C_6_-HSL and incubated at 25 °C for 1 h. CK means controls of PBS with the same amount of C_6_-HSL. The residual AHL was detected using strain *C. violaceum* CV026. (**c**) HPLC analysis of C_10_-HSL degradation. CK means controls of PBS with the same amount of C_10_-HSL; hydrolysis assay (AiiK: 4 μg/mL, C_10_-HSL: 50 μM) was carried out at 25 °C for 10 min, and then SDS was used to terminate the reaction; acidified controls assay (AiiK: 4 μg/mL, C_10_-HSL: 50 μM) was performed at 25 °C for 10 min, and SDS was used to terminate the reaction. The mixture was acidified to pH 2 by adding concentrated HCl and incubated at 25 °C overnight without agitation. The elution time of C_10_-HSL was at 6.57 ± 0.01 min and C_10_-HSL was monitored at 210 nm. All experiments were carried out in triplicates.

The *aiiK* gene was cloned from *K. huakuii* LAM0618^T^ by using the primers *aiiK*R and *aiiK*F, the full-length 837 bp gene, which encoded a 278-amino acid polypeptide belonging to putative metallo-β-lactamase. No signal peptide was detected in the intact amino acid sequence of AiiK by the analysis of SignalP 4.1. The *aiiK* gene was inserted into expression plasmid pET32a and the recombinant plasmid pET32a-*aiiK* was transformed into *E. Coli* BL21 (DE3). The recombined AiiK was expressed by the inducing of isopropyl-β-D-thiogalactopyranoside (IPTG) and then purified by using a Ni-NTA column. The molecular weight of the purified fusion protein was approximately 50 kDa, as determined by sodium dodecyl sulfate polyacrylamide gel electrophoresis (SDS-PAGE) analysis (Fig. 1); this vaule was much larger than the predicted molecular weight of 31.76 kDa because the purified recombinant AiiK contained complex soluble tags (Trx-Tag, His-Tag and S-Tag). A commonly conserved domain (HXHXDH-H-D) in the AHL lactonases has been detected in the amino acid sequence of AiiK. The results of *vivo* bioassays suggested that *E. coli* BL21 (DE3) cells harboring pET32a-*aiiK* has the ability to degrade N-Hexanoyl-L-homoserine lactone (C_6_-HSL) with the decreasing of purple pigment violacein’s diameter (Fig. 2a), and the results of *vitro* bioassays elucidated that AiiK can degrade 10 μM C_6_-HSL within 1 h at 25 °C by the disappearance of purple pigment violacein in comparison to the CK (Fig. 2b). The enzyme assays have confirmed that the AiiK was capable of degrading AHLs, including C_6_-HSL, N-(3-oxohexanoyl)-L-homoserine lactone (3-Oxo-C_6_-HSL), N-decanoyl-L-homoserine lactone (C_10_-HSL), and N-tetradecanoyl-L-homoserine lactone (C_14_-HSL) (data not shown). The opened lactone ring of AHL, which was catalyzed by AHL lactonase, was acidified at a low pH and the re-lactonization of the opened ring will be taken place resulting in an intact AHL^18–20^. Hydrolysis assay (Fig. 2c) and acidified control assay (Fig. 2c) suggested that the opened lactone ring of C_10_-HSL catalyzed by AiiK was re-lactonized when acidified at pH 2 over night, which indicated that AiiK was an AHL lactonase (Fig. 2c). And the way that hydrolysis assay and acidified control assay contributes a novel method to identifying AHL lactonase just by utilizing high-performance liquid chromatography (HPLC) analysis instead of using tandem mass spectrometry (MS). These results demonstrated that *aiiK* encodes an AHL lactonase belonging to AHL-degrading enzymes functioning as QQ enzymes. Hence, we named this gene the autoinducer inactivation gene from *K. huakuii* LAM0618^T^ (*aiiK*), which is the first autoinducer inactivation gene identified from the genus *Kurthia*.

### Kinetic properties of AiiK

To evaluate the substrate specificity, kinetic constants of AiiK for degrading different AHLs ranging from the number of carbon atoms to the carbonylation at the C-3 position of the *N*-acyl chain were performed by using a series of different concentrations of substrates and corresponding calculated velocities. GraphPad Prism 5 software was utilized to calculate the *K*_*m*_ and *k*_*cat*_ values by quoting the Michaelis-Menten equation. The *k*_*cat*_ values for all substrates ranged from 0.038 s^−1^ to 1.321 s^−1^ and the *K*_*m*_ values of recombinant AiiK ranged from 0.036 mM^−1^ to 0.315 mM^−1^ (Table 1), demonstrating AiiK can hydrolyze varieties of AHLs.

**Table 1:** Kinetic constants of AiiK for degrading AHLs.

AHL	Mean ± SD		*k*_*cat*_/*K*_*m*_(s^−1^·mM^−1^)
	*k*_*cat*_ (s^−1^)	*K*_*m*_(mM^−1^)	
C_6_-HSL	0.649 ± 0.069	0.204 ± 0.031	3.2
3-Oxo-C_6_-HSL	0.168 ± 0.026	0.066 ± 0.020	2.7
C_10_-HSL	1.321 ± 0.214	0.315 ± 0.065	3.9
C_14_-HSL	0.038 ± 0.002	0.036 ± 0.004	0.92

Note: Reactions were performed at pH 7.4 and 25 °C. And all data in this study were exbihited as mean ± the standard deviation (SD).

### Characterization of AiiK

**Figure 3:**
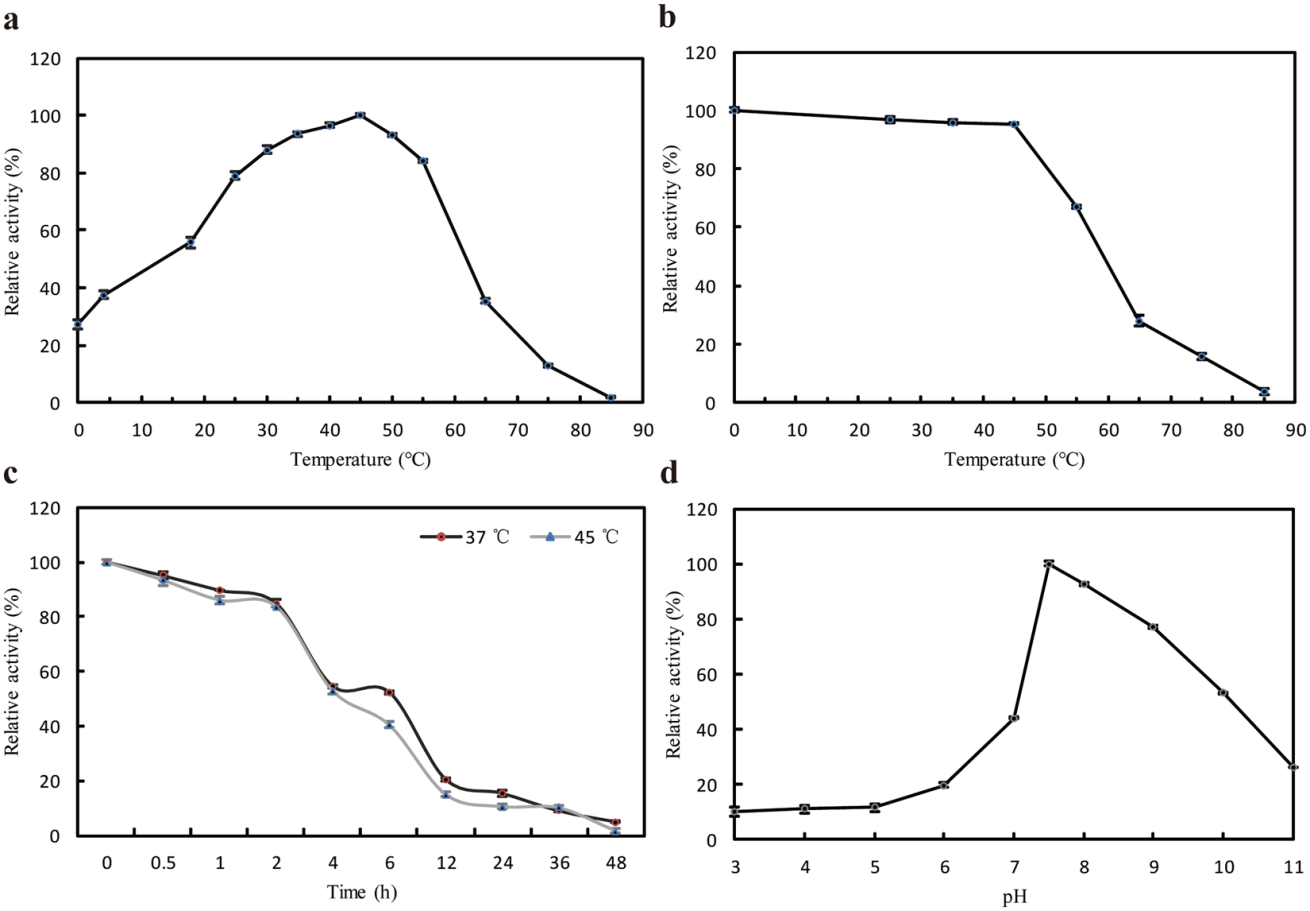
Characterization of AiiK. (**a**) Optimum temperature for enzyme activity. (**b**) Temperature stability of AiiK. (**c**) Thermostability of AiiK at 37 °C and 45 °C. (**d**) Effect of pH on AiiK. All experiments were carried out in triplicates.

The effects of temperature, pH, metal ions, and EDTA on AiiK were detected using C_10_-HSL as substrate. The optimal temperature of AiiK for degrading C_10_-HSL was 45 °C. AiiK maintained relatively high activity at tenperatures ranging from 18 °C to 55 °C and, interestingly, AiiK retained 27% of the maximum activity at 0 °C (Fig. 3a). In terms of thermostability, AiiK possessed more than 95% activity when the temperature was lower than 45 °C, and the relative activity significantly declined when the temperature exceeded 45 °C. However, AiiK still displayed 28% activity after a preincubation at 65 °C for 30 min (Fig. 3b). AiiK also exhibited approximately 20% activity after 12 h of preincubation at 37 °C and 45 °C (Fig. 3c). The optimal pH of AiiK for degrading C_10_-HSL at 25 °C was 7.5 (Fig. 3d). Low C_10_-HSL-degrading activity was detected when the pH was below 6, and AiiK maintained more than 53% activity when the pH level ranged from 7 to 10 (Fig. 3d). AiiK was stable at pH values ranging from 6 to 8, maintaining over 70% activity after preincubations for 30 min at 25 °C (data not shown).

**Figure 4:**
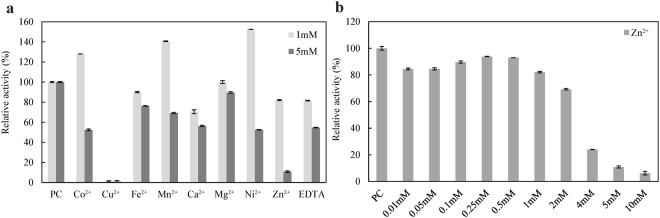
(**a**) Effect of metal ions and EDTA on AiiK activity. (**b**) Effect of zinc on AiiK activity *in vitro*. All experiments were carried out in triplicates. (PC stands for positive control, containing 4 μg/mL AiiK, 50 μM C_10_-HSL, and 10 mM PBS (pH 7.4), then the reaction was performed at 45 °C for 10 min).

AiiK activity increased with the addition of 1 mM Co^2+^, Mn^2+^, Mg^2+^, and Ni^2+^, with the most notable stimulation up to 152% with the addition of 1 mM Ni^2+^ (Fig. 4a). With the concentration of metal ions reaching at 5 mM, AiiK activity was all reduced. In addition, AiiK activity was extremely abolished in the presence of Cu^2+^ (Fig. 4a), which parallels MomL from *Muricauda olearia* Th120^14^. Moreover, AiiK activity decreased by approximately 19% and 45% after incubations with 1 and 5 mM EDTA, respectively (Fig. 4a). The effect of zinc on AiiK showed that a slight increase in activity of AiiK was observed with the increasing of Zn^2+^ concentration, then a maximum activity between 0.25 mM and 0.50 mM Zn^2+^, and a significant decrease was exhibited when the Zn^2+^ concentration exceeded 2.0 mM (Fig. 4b).

**Figure 5:**
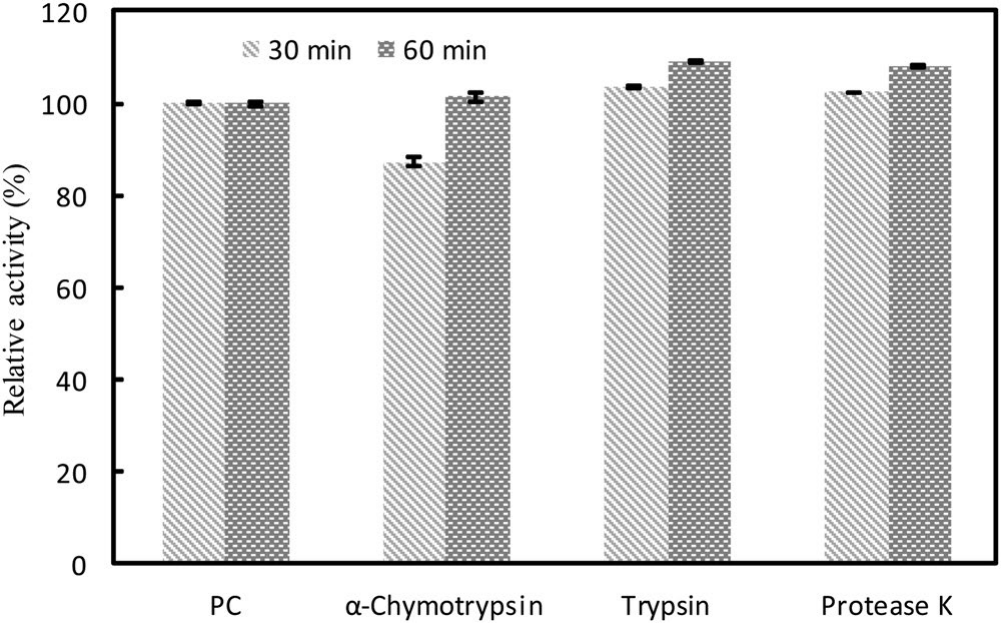
Resistance of AiiK to proteolysis. All experiments were carried out in triplicates. PC stands for positive control, containing 4 μg/mL AiiK, 50 μM C_10_-HSL, and 10 mM PBS (pH 7.4), then the reaction was carried out at 45 °C for 10 min.

The resistance of AiiK to α-chymotrypsin, trypsin, and protease K was detected using C_10_-HSL as substrate. AiiK maintained 87% and 101% activity after preincubations with α-chymotrypsin at 37 °C for 30 min and 60 min, respectively (Fig. 5). AiiK activity was enhanced up to 109% after preincubations with trypsin and protease K at 37 °C for 30 and 60 min (Fig. 5). It’s very interesting that AiiK exhibits higher activity when preincubated with these proteases at 37 °C for 60 min than that of the same condition for 30 min; this activity is better than observed for AiiA_AI96_^21^. Therefore, these results suggested that AiiK exhibited great protease-resistance.

### AiiK’s application in QQ on *P. aeruginosa* PAO1

**Figure 6:**
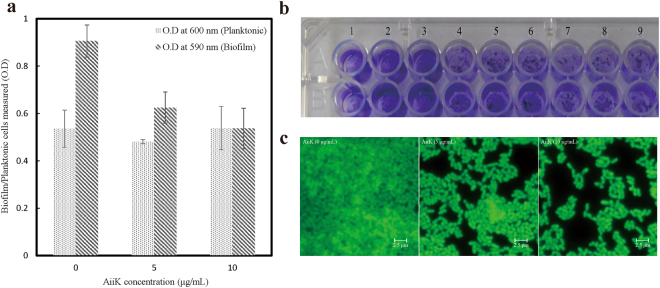
Effect of AiiK on biofilm formation in *P. aeruginosa* PAO1. (**a**) Biofilm formation by *P. Aeruginosa* PAO1 represented by crystal violet staining and planktonic cells of *P. Aeruginosa* PAO1. (**b**) The crystal violet staining of biofilm formed by *P. aeruginosa* PAO1. Lanes 1–3: 0 μg/mL of AiiK; Lanes 4–6: 5 μg/mL of AiiK; Lanes 7–9: 10 μg/mL of AiiK. (**c**) Fluorescence microscopy for visualization of biofilm formation by *P. aeruginosa* PAO1 stained with acridine orange.

To assess the effect of AiiK on biofilm formation in *P. aeruginosa* PAO1, assays were carried out in a 96-well microtiter plate. Biofilm cells were stained with crystal violet to evaluate the total intact biomass in the microtiter plate under the same experimental conditions. It was found that the inhibition rate of biofilm formation was enhanced with the increasing of application amount of AiiK (Fig. 6a and b) and AiiK did not kill *P. aeruginosa* PAO1from the O.D at 600 nm (Fig. 6a). *P. aeruginosa* PAO1 biofilm formation was visualized by fluorescence microscopy, indicating significant suppression of biofilm formation on special glass slides compared with the control (Fig. 6c).

**Figure 7:**
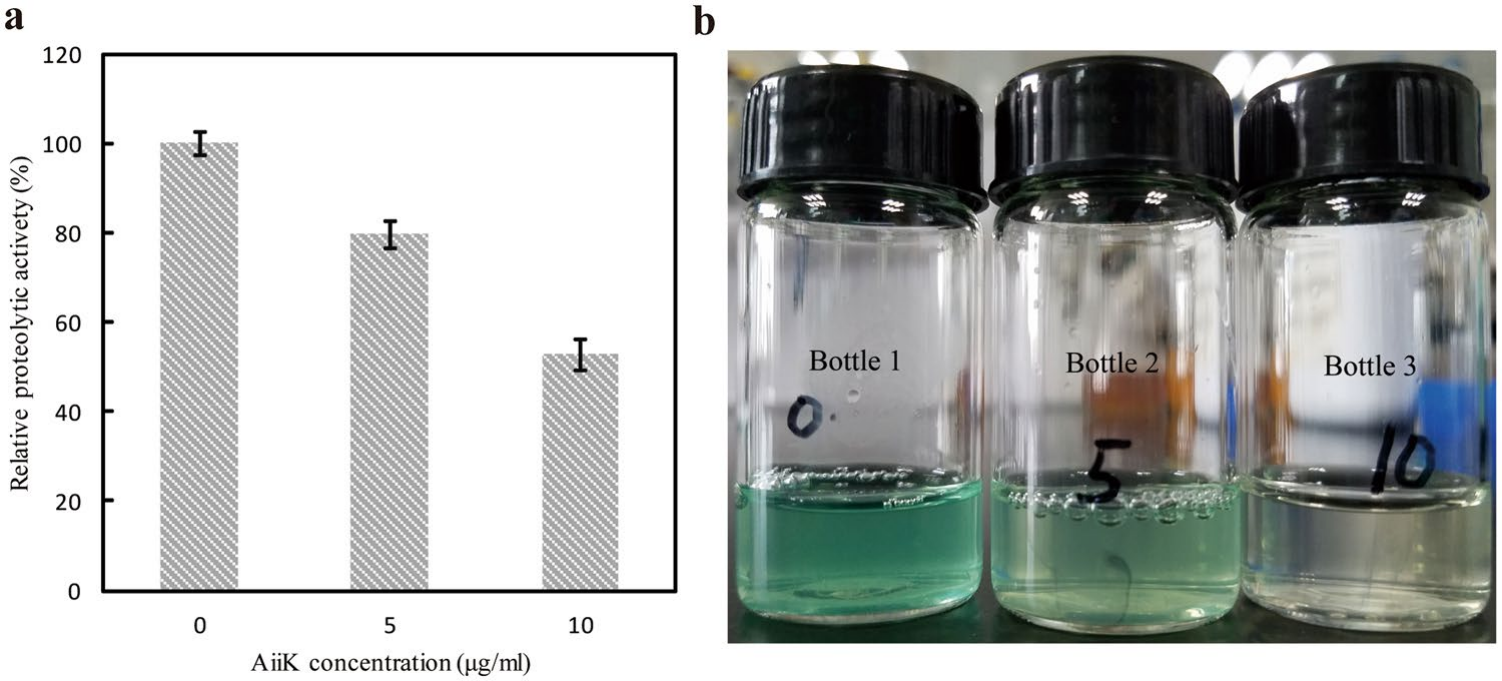
Effect of AiiK on virulence factor production. (**a**) The extracellular proteolytic activity of *P. aeruginosa* PAO1. (**b**) The pyocyanin production of *P. aeruginosa* PAO1. Bottle 1: 0 μg/mL of AiiK; Bottle 2: 5 μg/mL of AiiK; Bottle 3: 10 μg/mL of AiiK. All experiments were carried out in triplicates.

It was determined whether AiiK affected extracellular proteolytic activity in *P. aeruginosa* PAO1 and the results declared that 10 μg/mL AiiK reduced extracellular proteolytic activity by 47.3% after incubation with *P. aeruginosa* PAO1 at 37 °C for 12 h (Fig. 7a). Moreover, pyocyanin production of *P. aeruginosa* PAO1 was significantly decreased with the increasing of application amounts of AiiK (Fig. 7b and Table 2). Therefore, these results demonstrated that AiiK obviously inhibited the biofilm formation as well as the production of virulence factors consisting of extracellular proteolytic activity and pyocyanin production in *P. aeruginosa* PAO1, which contributes to the vital role of AiiK in QQ on *P. aeruginosa* PAO1.

**Table 2:** Effect of AiiK on pyocyanin production in *P. aeruginosa* PAO1.

AiiK concentration (μg/mL)	OD_520_	pyocyanin production (μg/mL)
0	0.223 ± 0.013	3.807 ± 0.222
5	0.207 ± 0.011	3.534 ± 0.188
10	0.064 ± 0.007	1.092 ± 0.120

## Discussion

**Figure 8:**
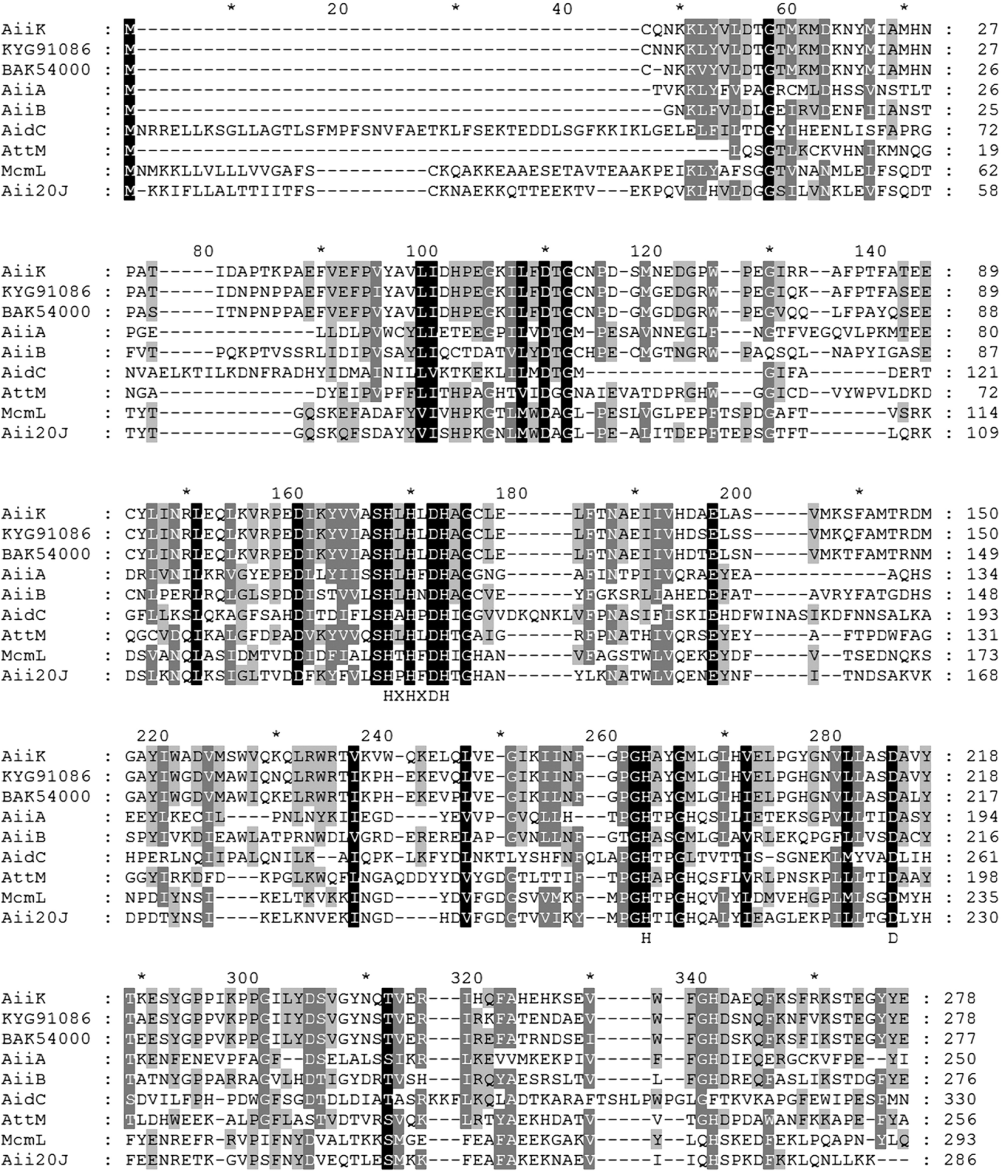
Multiple sequence alignment of amino acid sequences of AiiK (WP_029500404) and other representative AHL lactonases. The amino acid sequences were retrieved from NCBI or UniProt database. Sequence alignment was assesses by using of the MEGA 4.0 and GeneDoc. AiiK homologue from *Bacillus* sp. KCTC 13219 (KYG91086) and *Solibacillus silvestris* (BAK54000) showed the highest score and second highest score by BLAST searching in NCBI database. Other amino acid sequences of AHL lactonase are the first enzyme AiiA from *Bacillus* sp. strain 240B1 (AAF62398), AiiB (NP 396590) and AttM (AAD43990) from *Agrobacterium fabrum* str. C58, AidC from *Chryseobacterium* sp. strain StRB126 (BAM28988), MomL from *Muricauda olearia* (AIY30473), and Aii20J (AKN24544) from *Tenacibaculum* sp. 20J. These lactonases share a conserved domain (HXHXDH-H-D) essential for AHLs degradation.

Homology analysis based on the identified AHL lactonases at the level of amino acid sequence showed that AiiK shares only 26% identity with AiiA, the first enzyme identified capable of AHL degradation, from the strain *Bacillus* sp. 240B1^4^ (Fig. 8). When assessing similarity with other AHL lactonases, it was found AiiK shares 42% identity with AiiB from *Agrobacterium fabrum* str. C58^22^, 26% identity with AidC from *Chryseobacterium* sp. StRB126^23^, 31% identity with AttM from *A. fabrum* str. C58^24^, 23% identity with MomL from *Muricauda olearia*^14^, and 26% identity with Aii20J from *Tenacibaculum* sp. 20J^25^ (Fig. 8). The amino acid sequences of these AHL lactonases were retrieved from the NCBI or UniProt database. Sequence alignment was assessed using the MEGA 4.0 and GeneDoc. Compared with these AHL lactonases, the AHL-degradation activity of AiiK from *K. huakuii* LAM0618^T^ involves a conserved domain (HXHXDH-H-D) essential for QQ activity^4^ (Fig. 8). The HXHXD motif is essential to zinc-binding^14,26,27^; therefore, we studied the effect of zinc on AiiK *in vitro* and the effect of zinc on AiiK was similar to that on AidC^28^.

Analysis of the kinetic properties and characterization of AiiK revealed that AiiK exhibited a variable substrate spectrum and an efficient degradation of the AHL compounds used in this study, regardless of the number of carbon atoms of the *N*-acyl chain contained and whether there is carbonylation at the C-3 position. AiiK exhibited higher efficiencies at degrading C_6_-HSL and C_10_-HSL than 3-Oxo-C_6_-HSL and C_14_-HSL, demonstrating that AiiK presents a slightly preference for middle-long chain AHLs and is better at degrading unsubstituted AHLs at C-3 position than those with an oxo-substitution. In this study, C_10_-HSL was the optimal substrate for AiiK at 25 °C and pH 7.4. Furthermore, only 4 μg/mL AiiK was required to completely hydrolyze 30 μM C_10_-HSL within only 10 min at 25 °C compared to 20 μg/mL Aii20J from *Tenacibaculum* sp. 20J, which could hydrolyze only 10 μM C_10_-HSL in 20 min at 22 °C^25^. The optimal temperatures for AiiA_SS10_^29^, AiiA_B546_^30^, and AidC^23^ to degrade AHLs ranged from 20 °C to 30 °C. This range was lower than that for MomL (40 °C)^14^, AiiK (45 °C, this study), and AiiM (50 °C)^10^. In terms of thermostability, AiiK displayed 28% and 16% activity after preincubations at 65 °C and 75 °C for 30 min, respectively. Meanwhile Aii20J retained approximately 20% activity after a preincubation at 60 °C for 10 min as well as MomL maintaining approximately 18% activity after a preincubation at 80 °C for 30 min^14,25^. There are few cold-adapted AHL lactonases reported before, AiiK retained cold-adaption performance with 27% of the maximum activity at 0 °C.

As a common opportunistic pathogen, *P. aeruginosa* can cause severe diseases in plants and mammals, particularly in humans^31,32^. The virulence factors of *P. aeruginosa* associated with QS include pyocyanin production, extracellular protease secretion, and biofilm formation, which cause complicated biofilm associated infections and stimulate drug resistance^33^. These processes of QS are under the control of AHLs in *P. aeruginosa* reminds us that AHL lactonases can play a vital role in quenching these effects^34^. AiiK was demonstrated to be an AHL lactonase with a wide substrate spectrum and efficient degradation of the AHL compounds as well as AiiK remained approximately 20% activity after 12 h of incubation at 37 °C and was resistant to proteases, including α-chymotrypsin, trypsin, and protease K at 37 °C, providing the basis for experimental research by using AiiK to inhibit processes of QS in *P. aeruginosa* PAO1. The great protease-resistance and stability at 37 °C of AiiK play a vital role in the application of AiiK on QQ in *P. aeruginosa* PAO1, because the strain *P. aeruginosa* PAO1 could secrete proteases by QS when cultured at 37 °C. Our research confirmed that AiiK did display great abilities of suppressing the biofilm formation, production of pyocyanin, extracellular proteolytic activity in *P. aeruginosa* PAO1 rather than killing *P. aeruginosa* PAO1 while the progress of biofilm formation, indicating this is a promising anti-pathogenic strategy by which to control these pathogenic bacteria and to prevent antibiotic resistance. Therefore, these abilities of AiiK enpower a more valuable approach by quenching virulence factors and biofilm formation for controlling the biofilm-related infections caused by these pathogenic bacteria and expecting to be an anti-pathogenic drug.

In our study, the *aiiK* gene was the first autoinducer inactivation gene identified from the genus *Kurthia*. We firstly used hydrolysis assay and acidified control assay to confirm that AiiK was an AHL lactonase, which contributes to a novel method of identifying the AHL lactonases instead of using tandem MS. AiiK clearly quenched the effects of QS in *P. aeruginosa* PAO1 and did exhibit great characteristics such as good thermostability, cold adaptation, variable substrate spectrum, efficient degradation of AHLs, and great protease-resistance enable a wide application of AiiK, especially applied as a biocontrol agent or an anti-pathogenic drug. However, a detailed study about potential pharmaceutical application is still needed to investigate.

## Materials and Methods

### Bacterial strains, growth conditions, and chemicals

Selected bacterial strains and plasmids used in this study are listed in Table 3. The strains *K. huakuii* LAM0618^T^ and *P. aeruginosa* PAO1 were cultured in tryptic soy broth (TSB; Nippon Becton Dickinson, Tokyo, Japan) at 30 °C and 37 °C, respectively. *E. coli* DH5α and BL21 (DE3) strains were propagated in Luria-Bertani (LB) medium at 37 °C and appropriate antibiotic was added when required (final concentration of 100 μg/mL ampicillin). Reporter strain *C. violaceum* CV026 was fostered in LB medium containing 50 μg/mL kanamycin at 30 °C. The biofilm formation of *P. aeruginosa* PAO1 was tested on a M9 minimal media supplemented with 0.4% (w/v) glucose. Pseudomonas broth (PB, 20 g of Bacto-Peptone (Difco), 10 g of K_2_SO_4_, and 1.4 g of MgCl_2_) is a medium to promote the production of pyocyanin in liquid culture. And B-broth medium (5 g of yeast extract (Difco), 1 g of glucose, 10 g of casein hydrolysate 140 (Gibco), 5 g of NaCl and 1 g of K_2_HPO_4_, pH 7.3) was used for extracellular proteolytic activity assay of *P. aeruginosa* PAO1. C_6_-HSL, 3-oxo-C_6_-HSL, C_10_-HSL, and C_14_-HSL were purchased from Sigma-Aldrich (St. Louis, MO). All of these substrates were dissolved and prepared in chromatographic-grade methanol.

**Table 3:** Bacterial strains and plasmids.

Strain or plasmid	Description	Reference or source
Strains		
*Kurthia huakuii* LAM0618^T^	Wild type	ACCC 06121
*Escherichia coli* DH5α	λ^−^ф80d*lac*ZΔM15 Δ (*lacZYA-argF*) *U169 recA1 endA hsdR17* (r_K_^−^ m_K_^−^) *supE44 thi-1 gyrA relA1*	Tiangen
*Escherichia coli* BL21 (DE3)	F– *omp*T *hsd*S_B_ (r_B_–, m_B_–) *dcm gal* λ (DE3) pLysS Cm^r^	Tiangen
*Chromobacterium violaceum* CV026	ATCC 31532 derivative, *cviI*::Tn*5xylE* Km^r^, Sm^r^	From Dr. Guishan Zhang
*Pseudomonas aeruginosa* PAO1	Wild type	From Dr. Haijin Xu
Plasmids		
pET32a	Cloning vector, Amp^r^	Novagen
pET32a-*aiiK*	pET32a containing *aiiK* gene	This study

### Cloning of the *aiiK* gene from *K.huakuii* LAM0618^T^

A bacterial genome extraction kit (Tiangen, China) was used to extract the genomic DNA of *K. huakuii* LAM0618^T^, and this genomic DNA was applied as the template for PCR amplification. The *aiiK* coding region of the LAM0618^T^ genome was amplified using FastPfu DNA polymerase (TransGen Biotech, Beijing, China) with primers *aiiK*R (5′-CCGGAATTCATGTGTCAAAATAAAAAGTTGTAC-3′) and *aiiK*F (5′-CCCAAGCTTTTATTCGTAATACCCTTCCGTTGA-3′) that containing *EcoR*I and *Hind*III restriction sites (underlined). The PCR was carried out in accordance with the following cycling parameters: 94 °C for 5 min, 94 °C for 30 s, 55 °C for 30 s, and 72 °C for 1 min for 31 cycles, followed by 72 °C for 10 min. Both PCR products and pET-32a vector were digested with endonucleases *EcoR*I and *Hind*III, and then the digested PCR products were linked (at 25 °C for 1 h) onto the *EcoR*I-*Hind*III sites of pET32a through T4 DNA ligase for contribution to expression plasmid pET32a-*aiiK*. The nucleotide sequence was sequenced by Life Technologies Company (China).

### Expression and purification of recombinant AiiK

*E. coli* BL21 (DE3) cells harboring pET32a-*aiiK* were inoculated into 250 mL of fresh LB medium containing ampicillin at 37 °C and 180 rpm until an optical density at 600 nm of 0.6 was reached (approximately 3 h). Afterward, 0.5 mM IPTG was added to the culture medium, and then the cultures were incubated at 25 °C with moderate shaking (120 rpm) for 12 h. After incubation, the cultures were centrifuged at 10,000 rpm at 4 °C to harvest the *E. coli* BL21 (DE3) cells, and the cells were resuspended with lysis buffer (300 mM NaCl, 10 mM imidazole, and 50 mM Tris-HCl, pH 8.0). Lysozyme (final concentration of 1 mg/mL) and Benzonase nuclease (final concentration of 20 U/mL) (QIAGEN, Germany) were added to the lysis buffer, followed by incubation on ice batch for 45 min with moderate shaking. The suspension mixture was sonicated on ice batch for 15 min and then centrifuged at 10,000 rpm for 20 min at 4 °C to remove the cell debris. Supernatants were loaded onto Ni-NTA (QIAGEN, Germany), resulting in target protein bound to Ni-NTA which was later washed at least twice with wash buffer (30 mM imidazole, 50 mM Tris-HCl, and 300 mM NaCl, pH 8.0). AiiK was eluted using elution buffer (300 mM imidazole, 50 mM Tris-HCl, and 300 mM NaCl, pH 8.0). Subsequently, NaCl and imidazole were removed from the eluted fraction by ultrafiltration (molecular weight cutoff of 10 kDa) with 10 mM phosphate buffer saline (PBS, pH 7.4)^35^. SDS-PAGE and BAC protein assay kit (Shenerg Biocolor, Shanghai) were used to measure the purity and concentrations of purified recombinant AiiK, respectively. The purified recombinant AiiK protein (AiiK) was filtration sterilization by using 0.22 μm filters and then mixed with glycerol for storage at −20 °C for further study.

### AHL-degrading activity bioassays and enzyme assays

Reporter strain *C. violaceum* CV026 was endowed with the ability of evaluating AHL-degrading activity by producing the purple pigment violacein induced by short-chain AHLs^36^. 1 mL culture of strain *C. violaceum* CV026 incubated at 30 °C and 180 rpm for 16 h was mixed with 24 mL LB agar (1.6%) medium and poured in the plates. A 5.5 mm sterile paper disk was placed on an agar plate, and the AHL samples were applied on paper disk. Bioassay plates were incubated at 30 °C for 16 h, and then the appearance of pigment was detected. For *in vivo* bioassays, *E. coli* BL21 (DE3) cells harboring pET32a-*aiiK* were precultured into 5 mL of fresh LB medium containing ampicillin and 0.5 mM IPTG at 37 °C with rotary shaking at 180 rpm for 12 h. The culture was inoculated at a ratio of 2% into a new 5 mL LB medium containing ampicillin and 10 μM C_6_-HSL, then incubated at 37 °C for 0 h, 4 h, 6 h, 8 h, respectively. After incubation of different time, the culture supernatants were collected by centrifugation and used for the above-mentioned AHL-degrading activity bioassays. For *in vitro* bioassays, purified AiiK (final concentration of 4 μg/mL) was mixed with 10 μM C_6_-HSL, incubated at 25 °C for 1 h, and then used for the AHL-degrading activity bioassays immediately.

For the hydrolysis assay, the reaction mixture (500 μL) contained 5 μL AiiK (final concentration of 4 μg/mL), 50 μM AHLs (C_6_-HSL, 3-Oxo-C_6_-HSL, C_10_-HSL, or C_14_-HSL), and 10 mM PBS (pH 7.4). 2% SDS (m/v) was utilized to terminate the reaction after the mixture was incubated at 25 °C for 10 min. The remaining AHLs were then extracted three times using isopycnic ethyl acetate. Subsequently, ethyl acetate was evaporated with nitrogen flux at 25 °C, and the remaining AHLs were redissolved in 500 μL of methanol for HPLC analysis and quantification. For the control, which was handled and extracted via hydrolysis assay, comprised AiiK replaced with 10 mM PBS plus the same amount of AHLs. Furthermore, the acidified control assay, which contained the same amount of AiiK and AHLs, was performed at 25 °C for 10 min and terminated by 2% SDS. The mixture was acidified to pH 2 by adding concentrated HCl and incubated at 25 °C overnight without agitation, and then extracted following the hydrolysis assay.

An Agilent Technologies 1260 series HPLC system was applied to analyze and quantify AHLs degradation. The remaining AHLs were separated in an Agilent ZORBAX Eclipse Plus C18 column at 22 °C with a constant flow rate of 0.7 mL/min in isocratic elution and then detected at 210 nm. Eution procedure consisted of an isocratic profile of acetonitrile/water (27:73, v/v) for C_6_-HSL, acetonitrile/water (30:70, v/v) for 3-Oxo-C_6_-HSL, acetonitrile/water (60:40, v/v) for C_10_-HSL, and acetonitrile/water (80:20, v/v) for C_14_-HSL.

### Kinetic assay of AiiK activity

In order to determine the kinetics of AiiK for AHLs degradation, different concentration (25–150 μM, with an interval of 25 μM) of C_6_-HSL, 3-Oxo-C_6_-HSL, C_10_-HSL, or C_14_-HSL were added to PBS within AiiK (4 μg/mL). After this reaction was carried out at 25 °C for 10 min, the following procedure was based on the descriptions in enzyme assays section. The remaining AHLs were quantified by calculating the peak areas for a given retention time compared to those AHLs solutions of known concentrations via the analysis of HPLC. Furthermore, all assays were carried out in triplicates and AiiK replaced by 10 mM PBS plus the same amount of AHLs made up the control.

### AiiK characterization

Enzymatic properties of AiiK were characterized based on enzyme assays with 50 μM C_10_-HSL as the substrate. The optimal reaction temperature of AiiK was determined by evaluating activity at temperatures from 0 °C to 85 °C (0 °C, 4 °C, 18 °C, 25 °C, 30 °C, 35 °C, 40 °C, 45 °C, 50 °C, 55 °C, 65 °C, 75 °C, and 85 °C). To evaluate the thermostability of AiiK, the residual activity of AiiK was measured after preincubations at different temperatures (25 °C, 35 °C, 45 °C, 55 °C, 65 °C, 75 °C, and 85 °C) for 30 min. The optimal reaction pH of AiiK was estimated after incubations in a variety of pH levels, ranging from 2 to 11 (Na_2_HPO_4_/citric acid for pH 2.0 to 7.0, Tris-HCl for pH 8.0 to 9.0, and Gly/NaOH for pH 10.0 to 11.0), for 10 min at 25 °C. The pH stability of AiiK was performed via detecting the residual activity of AiiK after it was preincubated at different pH levels for 30 min at 25 °C.

The effects of metal ions and EDTA on AiiK were determined by preincubation with 1 mM and 5 mM concentrations of various metal ions (Co^2+^, Cu^2+^, Fe^2+^, Mn^2+^, Ca^2+^, Mg^2+^, Ni^2+^, and Zn^2+^) and EDTA at 4 °C for 30 min. The effect of zinc on AiiK was detected by preincubation with various concentrations of Zn^2+^ (0.01 mM-10 mM) at 4 °C for 30 min. Subsequently, the residual activity of AiiK was measured at 45 °C for 10 min using the same method as that of the enzyme assays described above.

To study resistance of AiiK to proteolysis, AiiK was preincubated with α-chymotrypsin, trypsin, and protease K (at a ratio of 1:10, w/w; protease/AiiK) in 10 mM PBS (pH 7.4) at 37 °C for 30 min and 60 min. Subsequently, the residual activity of AiiK was measured at 45 °C for 10 min by using the same method as that of the enzyme assays described above. All experiments were carried out in triplicates and the detailed experiments were performed under the precondition of more than 20% of the C_10_-HSL had been remained.

### Effect of AiiK on biofilm formation in *P. aeruginosa* PAO1

A static microtiter plate assay, modified from the research of Cady *et al*.^37^ and Rajesh and Ravishankar Rai^38^, was used to test the effect of AiiK on biofilm formation in *P. aeruginosa* PAO1. Strain *P. aeruginosa* PAO1 was precultured in TSB overnight at 37 °C with rotary shaking at 150 rpm. After preincubation, the cultures were centrifugated at 10,000 rpm to harvest the *P. aeruginosa* PAO1 cells, and the cells were rinsed with sterile 10 mM PBS (pH 7.4) for three time. After the cells were resuspended in M9 minimal media with a concentration of 2 × 10^7^ cfu/mL (determined through optical density and plate count assay), the *P. aeruginosa* PAO1 inocula were then premixed with AiiK (0, 5, or 10 μg/mL). For control wells, 10 mM PBS was added into the inocula instead of AiiK. A total of 200 μL of these cells/AiiK mixtures were dispensed into a 96-well microtiter plate and then incubated at 37 °C for 24 h without shaking. Planktonic cells from the plate were transferred gently to a new 96-well microtiter plate to measure the optical density at 600 nm. The cells at the bottom of the 96-well microtiter plate were stained with 20 μL of 0.2% crystal violet at 25 °C for 15 min to estimate the quantity of biofilm formation. The wells were washed very gently with sterile distilled water three times until the crystal violet was removed. Finally, absorbance at 590 nm was detected after the crystal violet was extracted from the cells by adding 100 μL of 95% ethanol.

The effects of AiiK on biofilm formation in *P. aeruginosa* PAO1 were visualized and assessed as previously reported with minor modifications^38^. Briefly, the growth, resuspension and premixing with AiiK (0, 5, or 10 μg/mL) of *P. aeruginosa* PAO1 were performed via static microtiter plate assay, and control samples were not treated with AiiK. Subsequently, 2 mL of these cells/AiiK mixtures were dispensed into sterile PA bottles containing special sterile glass slides, and biofilm can grow well on the 5 × 5 mm special glass slides with incubation at 37 °C for 24 h without agitation. After incubation, the slides were dried and stained with 0.1% of acridine orange (Sigma, China) for 2.5 min. The biofilm cell attachment was observed under a fluorescence microscope (excitation at 490 nm and emission at 525 nm), and the inhibition of biofilm formation in AiiK-treated and control samples was compared visually.

### Effect of AiiK on extracellular proteolytic activity in *P. aeruginosa* PAO1

The effect of AiiK on extracellular proteolytic activity in *P. aeruginosa* PAO1 was determined as previously described with modifications^39^, where azocasein was used as substrate. *P. aeruginosa* PAO1, cultured at 37 °C for 12 h with rotary shaker at 150 rpm, was inoculated in B-broth containing different concentrations of AiiK (0, 5, or 10 μg/mL). The absorbance of the culture was measured at 590 nm. The supernatants were then utilized as crude extract to determine the extracellular proteolytic activity of the culture through centrifugation of the culture at 12,000 rpm. The reaction mixture (500 μL) for extracellular proteolytic activity assay contained 150 μL of crude extract and 250 μL of 2% azocasein dissolved in 10 mM PBS (pH 7.4) and was incubated at 37 °C for 30 min. A total of 1.2 mL of 10% trichloracetic acid was added to terminate the reaction. The mixture was then incubated at 25 °C for 15 min. After this incubation, 1.2 mL supernatants of the mixture, which were collected by centrifugation at 3,000 g for 5 min, was mixed with 1 mL of 1 M NaOH in a new tube, and the measurement of absorbance was then determined at 440 nm. Furthermore, the effect of AiiK on extracellular proteolytic activity in *P. aeruginosa* PAO1was measured as OD_415_/OD_590_.

### Effect of AiiK on pyocyanin production in *P. aeruginosa* PAO1

The effect of AiiK on pyocyanin production in *P. aeruginosa* PAO1 was detected as previously described with some modifications^40^. *P. aeruginosa* PAO1 was incubated in PB containing different concentrations of AiiK (0, 5, or 10 μg/mL) at 37 °C for 18 h with rotary shaker at 150 rpm. A total of 5 mL culture supernatants collected by centrifugation at 12,000 rpm were mixed with 3 mL of chloroform, and then the mixture was reextracted by adding 1 mL of 0.2 M HCl. Subsequently, the measurement of absorbance was determined at 520 nm, and the concentration of pyocyanin was estimated by multiplying the optical density at 520 nm (OD_520_) by 17.072.

### Data availability statement

The authors confirm that all data are available.
